# Coordinated changes of HIV-1 Gag and Env during assembly and maturation

**DOI:** 10.1186/1742-4690-10-S1-O2

**Published:** 2013-09-19

**Authors:** Hans-Georg Kräusslich

**Affiliations:** 1 Department of Virology, University of Heidelberg, D-69120 Heidelberg, Germany

## 

Formation of infectious HIV-1 particles involves assembly of Gag polyproteins into immature particles and subsequent assembly of mature capsids following proteolytic disassembly of the Gag shell. Maturation is essential for viral infectivity; it is required for full activity of viral enzymes, for virus uncoating and for efficient membrane fusion. Morphological maturation involves dramatic rearrangements in the interior of the virus, but also leads to coordinated clustering of the low number of Env trimers on the virion surface. Env incorporation is dependent on specific regions in Gag and the C-terminal tail of Env, but appears to be mediated by formation of Env patches larger than the actual assembly site. Here, we will discuss aspects of Gag-dependent Env recruitment and morphological alterations during virion maturation.

